# Associations between symptoms of attention-deficit/ hyperactivity disorder and life satisfaction in medical students: the mediating effect of resilience

**DOI:** 10.1186/s12909-018-1261-8

**Published:** 2018-07-13

**Authors:** Meng Shi, Li Liu, Xiao Sun, Lie Wang

**Affiliations:** 1 English Department, School of Fundamental Sciences, China Medical University, No. 77 Puhe Road, Shenyang North New Area, Shenyang, Liaoning Province People’s Republic of China; 2 Department of Social Medicine, School of Public Health, China Medical University, No. 77 Puhe Road, Shenyang North New Area, Shenyang, Liaoning Province People’s Republic of China; 3 Section of Sports, China Medical University, No. 77 Puhe Road, Shenyang North New Area, Shenyang, Liaoning Province People’s Republic of China

**Keywords:** ADHD symptoms, Life satisfaction, Resilience, Medical students

## Abstract

**Background:**

Research on symptoms of attention-deficit/hyperactivity disorder (ADHD) in medical students is rather scant. Studying the disorder in this population, especially its associations with positive psychological constructs can further the understanding of mental health in future physicians. The objectives of the present study were to investigate the prevalence of ADHD symptoms in medical students, to examine the relationships between ADHD symptoms and life satisfaction, and to explore the mediating role of resilience on the associations.

**Methods:**

This cross-sectional study was carried out at one medical university in China, in June 2016. Self-reported questionnaires consisting of Adult ADHD Self-Report Scale (ASRS), Wender Utah Rating Scale (WURS), Conner-Davidson Resilience Scale (CD-RISC), Satisfaction With Life Scale (SWLS), and socio-demographic characteristics, were distributed to the students. Hierarchical linear regression analyses were used to examine the effects of ADHD symptoms on life satisfaction, and asymptotic and resampling strategies were used to explore the mediating role of resilience.

**Results:**

A total number of 521 medical students became final subjects. Based on the cutoffs of the scales, 1.54% of the medical students were highly likely to have ADHD, and 6.91% of the students were likely to have ADHD. Only inattention was negatively correlated with life satisfaction in the students. Resilience functioned as a mediator in the relationship between inattention and life satisfaction.

**Conclusions:**

The prevalence of ADHD symptoms among Chinese medical students could be relatively high. Inattention is significantly related to life satisfaction among the students. Early identification of medical students with ADHD symptoms should be warranted. Resilience intervention programs might be undertaken to enhance life satisfaction in medical students, especially for those with inattention symptoms.

## Background

Attention-deficit/hyperactivity disorder (ADHD) is characterized by developmentally inappropriate symptoms of inattention and/or hyperactivity-impulsivity [1]. Although traditionally considered a childhood disorder, it is increasingly recognized that ADHD symptoms can persist into adulthood. The estimated prevalence of clinician assessed ADHD in adults is approximately 4% [2, 3]. Recent large-scale epidemiological studies reveal that the prevalence rate of ADHD symptoms among university students varies widely, ranging from 2.8 to 12.3%, and up to 22.8% of the students may demonstrate ADHD sub-threshold symptoms [4–7]. The chronic condition of ADHD has been consistently shown to be related to impairments across multiple domains of life, such as social relationships, occupational attainment, academic performance, substance use, and other psychiatric comorbidities [5, 8–11]. As of yet, however, very few studies have been conducted to examine the prevalence of ADHD symptoms among medical students. This forms a sharp contrast with the findings of a recent large survey in 145 U.S. medical schools, demonstrating that ADHD was the most common self-disclosed disability among all types of disabilities in medical students receiving accommodations, far above the rate of other psychological disabilities combined [12].

Although increasing attention has been paid to ADHD in adults, prior research has predominantly focused on negative effects accompanied by the disorder, whereas studies on associations between ADHD and positive outcomes, such as life satisfaction, are very limited. The only relevant study carried out among university students revealed that even mild ADHD symptoms were significantly correlated with lower levels of life satisfaction [13]. Compared with categorical approach, the dimensional approach of ADHD can capture more information for research and clinical practice [14]. Using a bifactor model, one study, conducted in middle-aged adults, showed that in comparison to hyperactivity, inattention was more strongly related to occupational, functional and psychological outcomes, including life satisfaction, even after adjustment for depression and anxiety [15]. However, to the best of our knowledge, no studies have been carried out in medical students to examine the associations of ADHD symptoms with life satisfaction.

Despite the related impairments induced by ADHD symptoms, some individuals may function well in their life. It is logical to infer that these people may possess certain positive psychological resources to survive and thrive in the face of obstacles they encounter. Positive psychology has been increasingly used in the prevention and treatment of mental health problems, as well as in the promotion of subjective well-being [16], and resilience is one of the positive psychological resources that people with ADHD symptoms may use to deal with challenges in life [17, 18]. Resilience fundamentally refers to the process of negotiating, managing and adapting to significant sources of stress and trauma [19]. The study by Wilmshurst et al. demonstrated that college students with a diagnosis of ADHD reported higher level of parental supports than controls and might represent an especially resilient group [20]. However, ADHD was shown to be associated with lower resilience in adolescents [21]. Meanwhile, resilience was found to be positively related to life satisfaction among medical students, accounting for up to 18% of its variance [22]. According to transactional model of stress and coping [23], resilience, a psychological strength and resource, can influence the secondary appraisal process of a stressor, whereby mediating the relationship between the stressor and its outcomes, but the roles that resilience might play on the associations between ADHD symptoms and life satisfaction have not yet been examined.

Given that ADHD has been revealed to rank the top self-disclosed disability among medical students [12], and extant research on ADHD symptoms in this population is extremely underrepresented, we conducted the present study with the following aims: 1) to investigate the prevalence of ADHD symptoms in Chinese medical students; 2) to examine the relationships between the two dimensions of ADHD symptoms and life satisfaction in medical students; 3) and to explore the possible mediating role of resilience in the relationships between ADHD symptoms and life satisfaction.

## Methods

### Study design and subjects

The cross-sectional study was carried out at China Medical University, in June 2016. Based on academic year, the stratified cluster sampling approach was used to recruit whole classes of clinical medicine students from English language classes. All the students were informed about the aims of the study and invited to voluntarily participate in the survey before the questionnaires were distributed. The study was approved by the Committee on Human Experimentation of China Medical University and informed consents were obtained from all the subjects.

### Measurement of current ADHD symptoms

The Chinese version of Adult ADHD Self-Report Scale (ASRS) was used to assess current ADHD symptoms among the students [24]. ASRS consists of two subscales, inattention and hyperactivity, each having 9 items. Sample items include “How often do you have trouble wrapping up the final details of a project, once the challenging parts have been done?” and “How often do you feel overly active and compelled to do things, like you were driven by a motor?”. The respondents were asked to rate each item on a 5-point Likert scale from 0 (never) to 4 (very often), based on their experiences over the past six months. Individuals with either subscale score of 24 or greater were considered highly likely to have ADHD, scores between 17 and 23 were classified likely, and scores from 0 to 16 were unlikely to have ADHD [25]. The Chinese version of ASRS has demonstrated sound reliability and validity in previous studies [4, 24, 26]. In this study, the Cronbach’s alpha coefficients for inattention dimension and hyperactivity dimension were 0.78 and 0.85 respectively.

### Measurement of childhood ADHD symptoms

Wender Utah Rating Scale (WURS) was adopted to assess childhood ADHD symptoms of the subjects [27]. The 25-item WURS was designed to retrospectively describe childhood ADHD symptoms (e.g. “As a child, I had concentration problems, easily distracted” and “As a child, I was acting without thinking, impulsive”). Each item is rated on a 5-point Likert scale from 0 (not at all or very slightly) to 4 (very much). A total score of 46 was used as cutoff point for having childhood ADHD [27]. The Chinese version of WURS has shown adequate psychometric properties [5, 28]. The Cronbach’s alpha was 0.94 in the present study.

### Classification criteria for ADHD symptomatic group and non-symptomatic group

For the diagnosis of adult ADHD, individuals should have both significant current ADHD symptoms and childhood ADHD symptoms. Thus, the subjects who had a WURS score ≥ 46 and at least one dimension of ASRS score ≥ 17 were classified as symptomatic group, whereas the remaining subjects belonged to non-symptomatic group.

### Measurement of resilience

Connor–Davidson Resilience Scale (CD-RISC) measures the ability to cope with stress and adversity [29]. The 25-item CD-RISC possesses high psychometric properties ratings among all resilience measurement scales [19]. Sample items include “I am able to adapt when changes occur” and “Under pressure, I stay focused and think clearly”. Each item is rated on a 5-point Likert scale from 0 (not true at all) to 4 (true nearly all the time). Higher score of the total scale indicates higher level of resilience. Due to its high reliability and validity, the scale has been used in many Chinese populations [30, 31]. The Cronbach’s alpha for CD-RISC in the present study was 0.94.

### Measurement of life satisfaction

Satisfaction With Life Scale (SWLS) was developed to measure global level of life satisfaction [32]. The scale consists of five items (e.g. “in most ways my life is close to my ideal” and “the conditions of my life are excellent”). Each item is answered on a 7-point Likert scale from 1 (strongly disagree) to 7 (strongly agree), with higher overall score indicating higher level of life satisfaction. Previous research has demonstrated satisfactory predictive validity and reliability of the scale among various age groups [22, 32, 33]. In the present study, the Cronbach’s alpha for SWLS was 0.91.

### Demographic characteristics

Demographic information regarding age, gender, and academic year were obtained in the study.

### Statistical analysis

All analyses were performed using SPSS 13.0. All statistical tests were two-sided and the significance level was set at *p* <  0.05. Chi-squared tests and t-tests were used to compare differences in categorical and psychological variables between ADHD symptomatic group and non-symptomatic group. Pearson’s correlation was used to examine correlations among inattention, hyperactivity, resilience and life satisfaction. Hierarchical regression analysis was used to explore the effects of groups of independent variables on life satisfaction. Standardized estimate (β), F, R^2^ and R^2^-changes (△R^2^) for each step were provided. Asymptotic and resampling strategies, developed by Preacher and Hayes [34], were used to examine the mediating role of resilience (a*b product) on the associations of inattention and hyperactivity with life satisfaction. The bootstrap estimate was based on 5000 bootstrap samples. The bias-corrected and accelerated 95% confidence interval (BCa 95%CI) for each a*b product was calculated, and a BCa 95%CI excluding 0 indicated a significant mediating role. All the continuous variables were standardized in order to avoid multicollinearity before the regression analyses were performed.

## Results

### Demographic characteristics of the subjects

The demographic characteristics of the subjects and the distributions of life satisfaction are shown in Table 1. Among the 560 clinical medicine students attending English language classes, 526 students returned questionnaires in class. 5 invalid questionnaires were excluded, and 521 students became the final subjects (effective response rate: 93.04%), which consisted of 202 first year students, 183 second year students, 64 third year students and 72 fourth year students. There were 341 female medical students and 180 male students. The age of the subjects ranged from 18 to 25 (M = 20.42, SD = 1.39).

**Table 1:** Demographic characteristics of the subjects in life satisfaction (*N* = 521)

Variables	N	%	Life satisfaction (Mean ± SD)
Age group			
18–20	306	58.73%	22.93 ± 7.08
21–25	215	41.27%	23.10 ± 6.87
Gender			
Male	180	34.55%	22.71 ± 6.81
Female	341	65.45%	23.16 ± 7.08
Academic year			
First year	202	38.77%	23.33 ± 7.00
Second year	183	35.13%	22.56 ± 7.21
Third year	64	12.28%	23.16 ± 6.24
Fourth year	72	13.82%	23.07 ± 7.07

Life satisfaction: Satisfaction With Life Scale

### Characteristics of the subjects in ADHD symptomatic group and non-symptomatic group

Among the 521 medical students, 47 students (9.02%) had a WURS score ≥ 46. 224 students (42.99%) had an inattention score between 17 and 23, and 16 students (3.07%) had a score ≥ 24, while 93 students (17.85%) had a hyperactivity score between 17 and 23, and 7 students (1.34%) had a score ≥ 24. Based on the cutoffs of WURS and ASRS, 8 medical students (1.54%) were highly likely to have ADHD, and 36 students (6.91%) were likely to have ADHD. These 44 students are categorized as ADHD symptomatic group.

The comparison of the characteristics of the subjects in ADHD symptomatic group and non-symptomatic group are presented in Table 2. The younger group had a slightly higher rate of ADHD symptoms than the older group (*p* = 0.049). There were no significant differences between the two groups in terms of gender (*p* = 0.208) and academic year (*p* = 0.258). The ADHD symptomatic group had significantly lower levels of resilience (*p* <  0.001) and life satisfaction (*p* <  0.001) relative to non-symptomatic group.

**Table 2:** Characteristics of the subjects in ADHD symptomatic group and non-symptomatic group (*N* = 521)

Variables	ADHD symptomatic N (%)	ADHD non-symptomatic N (%)	P
Age group			
18–20	32 (10.46%)	274 (89.54%)	0.049
21–25	12 (5.58%)	203 (94.42%)	
Gender			
Male	19 (10.56%)	161 (89.44%)	0.208
Female	25 (7.33%)	316 (92.67%)	
Academic year			
First year	21 (10.40%)	181 (89.60%)	0.258
Second year	17 (9.29%)	166 (90.71%)	
Third year	3 (4.69%)	61 (95.31%)	
Fourth year	3 (4.17%)	69 (95.83%)	
	Mean ± SD	Mean ± SD	
Resilience	57.50 ± 16.06	68.85 ± 14.01	< 0.001
Life satisfaction	18.00 ± 6.69	23.46 ± 6.84	< 0.001

Resilience: Connor–Davidson Resilience Scale; Life satisfaction: Satisfaction With Life Scale

### Pearson correlations among ADHD symptoms, resilience and life satisfaction

The means, standard deviations and correlations among ADHD symptoms, resilience and life satisfaction are shown in Table 3. As revealed in the table, both inattention and hyperactivity were negatively related to resilience (inattention: *r* = − 0.34, *p* <  0.01; hyperactivity: *r* = − 0.28, *p* < 0.01) and life satisfaction (inattention: *r* = − 0.27, *p* < 0.01; hyperactivity: *r* = − 0.21, *p* < 0.01). Resilience was moderately correlated with life satisfaction (*r* = 0.47, *p* < 0.01).

**Table 3:** Correlations among ADHD symptoms, resilience and life satisfaction

Variables	Mean	SD	1	2	3	4
1. Inattention	15.45	5.19	1			
2. Hyperactivity	11.58	5.67	0.66^**^	1		
3. Resilience	67.89	14.53	−0.34^**^	− 0.28^**^	1	
4. Life satisfaction	23.00	6.98	−0.27^**^	− 0.21^**^	0.47^**^	1

**p < 0.01 (two-tailed)

### Hierarchical regression results

The results of the hierarchical regression of life satisfaction are presented in Table 4. The two dimensions of ADHD symptoms, inattention and hyperactivity, accounted for 8% of the variance in life satisfaction. After controlling for age and gender, only inattention was significantly related to life satisfaction (β = − 0.23, *p* < 0.01).

**Table 4:** Hierarchical linear regression analyses results

Variables	Step 1 β (95%CI)	Step 2 β (95% CI))	Step 3 β (95% CI))
Step 1			
Age	−0.00 (− 0.09,0.09)	−0.02 (− 0.11, 0.06)	−0.02 (− 0.09, 0.06)
Gender	0.03 (− 0.12, 0.25)	0.02 (− 0.13, 0.22)	0.04 (− 0.08, 0.25)
Step 2			
Inattention		−0.23** (− 0.34, − 0.12)	−0.11* (− 0.21, − 0.00)
Hyperactivity		−0.06 (− 0.18, 0.05)	−0.02 (− 0.13, 0.08)
Step 3			
Resilience			0.42** (0.34, 0.51)
F	0.24	10.56**	31.24**
R^2^	0.00	0.08	0.23
△R^2^	0.00	0.08	0.16

*p < 0.05(two-tailed); **p < 0.01 (two-tailed)

The effect of resilience on life satisfaction was significantly positive (β = 0.42, *p* < 0.01), explaining up to 16% of the variance in life satisfaction.

### The mediating role of resilience in the relationships between ADHD symptoms and life satisfaction

The path coefficients, effect size of the mediator (a*b products), and BCa 95%CI of the products are displayed in Table 5. Because hyperactivity was not significantly related to resilience (a path) and life satisfaction (c path), resilience did not mediate the relationship between hyperactivity and life satisfaction in the students. Resilience significantly mediated the association of inattention with life satisfaction (a*b = − 0.120, BCa 95%CI: − 0.179, − 0.067, *p* < 0.01).

**Table 5:** Mediating role of resilience in the relationship between ADHD symptoms and life satisfaction

Predictors	Path coefficients				a*b (BCa 95%CI)
	c	a	b	c’	
Inattention	−0.228**	− 0.284**	0.424**	− 0.108*	−0.120** (− 0.179, − 0.067)
Hyperactivity	−0.064	− 0.093	0.424**	− 0.024	−0.039 (− 0.092, 0.014)

*p < 0.05 (two-tailed); **p < 0.01 (two-tailed). c: associations of ADHD symptoms with life satisfaction; a: associations of ADHD symptoms with resilience; b: associations of resilience with life satisfaction after controlling for the predictor variables; c’: associations of ADHD symptoms with life satisfaction after adding resilience as mediator

## Discussion

This is one of the few studies that focused on ADHD symptoms in medical students, and the first one to examine the associations of ADHD symptoms with life satisfaction in medical students, and the mediating effect of resilience on the associations. Although the proportion of medical students who were highly likely to have ADHD was rather low (1.54%), the study demonstrated that 8.45% of the students had symptomatic ADHD. This result was consistent with the prevalence of possible ADHD (8.7%) found among African medical students [35], which might suggest that ADHD symptoms could be largely under-recognized and ignored among vulnerable and stressful medical students compared to other psychological problems. Early identification of medical students with ADHD symptoms should be warranted and is the prerequisite to bring them for treatment. A systematic review has shown that without treatment, people with ADHD have poorer long-term outcomes in almost every aspect of life, such as self-esteem, social behaviors and occupational attainment, relative to those without ADHD [36]. Compared with non-symptomatic ones, the symptomatic medical students showed significantly lower level of resilience, which was consistent with other prior research [21]. One explanation is that ADHD is often comorbid with other psychiatric disorders, including depression and anxiety [3, 37], which are negatively related to resilience [38]. In addition, both inattention and hyperactivity are associated with substance use [11], which in turn is also associated with anxiety and depression [39].

One of the major findings of this study is that inattention was found to be significantly related to life satisfaction among medical students, which confirmed previous findings in other populations. Prior research conducted among middle school students showed that inattention was a consistent predictor of life satisfaction in both student self-ratings and teacher ratings of ADHD symptoms, whereas in neither ratings, hyperactivity was significantly associated with life satisfaction of the students [40]. Moreover, another study also revealed that inattention factor was significantly correlated with life satisfaction in middle-aged adults, while hyperactivity factor was not significantly related to their life satisfaction [15]. Two reasons may account for the results. Firstly, ADHD symptoms change with age. While hyperactivity decreases as people grow into their adolescence and adulthood, inattention continues to exist, becoming the dominant form of ADHD symptoms [41]. Secondly, compared to hyperactivity, inattention has been found to be more strongly associated with psychological distress, such as depression and anxiety [15], which in turn is negatively related to life satisfaction.

Another major finding of the present study is that resilience was shown to play a mediating role in the relationship between inattention and life satisfaction among the students. Inattention was not only directly related to life satisfaction, but also indirectly related to it through resilience. Higher scores on inattention among the medical students were associated with lower levels of resilience, which was correlated with lower levels of life satisfaction. In contrast, lower scores on inattention among the students were associated with higher levels of resilience, which was correlated with higher levels of life satisfaction. As positive psychology has been successfully used in the prevention and treatment of psychological distress, the results of the present study deserve attention in terms of ADHD treatment in medical students and life satisfaction promotion in those with ADHD symptoms. As a vital positive psychological resource and state-like construct, resilience is malleable and can be enhanced for each individual. A systematic review of resilience intervention programs in adults did show some degree of effectiveness despite large heterogeneity with regard to intervention approaches, formats and populations [42]. For example, it has been demonstrated that the level of resilience in college students is significantly improved after a four 2-h weekly intervention [16]. Some of the strategies used for cognitive-behavioral therapy in resilience intervention, such as cognitive restructuring and coping skills [43], are often incorporated into psychosocial treatment of ADHD, which has been proved to be an effective treatment option for adults with ADHD [44, 45]. Thus, medical schools can adopt evidence-based measures to examine the efficacy of tailored resilience intervention programs in medical student with ADHD. Our study confirmed that inattention was more prevalent in adults relative to hyperactivity. Inattention symptoms in adults are expressed as difficulty sustaining attention and finishing tasks, distractibility and forgetfulness, and poor time management [46], which are likely to affect academic performance as well as life satisfaction of the students. However, a high level of resilience in youth with ADHD is characterized by more family and peer social support, better psychosocial functions, and less internalizing disorders [18, 47], which are negatively related to life satisfaction. It should be noted that the variables included in the study only accounted for 23% of the variance in life satisfaction among the students. Several potential factors, such as personality traits, social factors, and other psychological factors, may affect their life satisfaction and were not included in the model.

Based on the framework of positive psychology, this study examined the associations of two dimensions of ADHD symptoms with life satisfaction in a relatively large sample of medical students with a high effective response rate, and also explored the mechanism linking the association. The limitations of the current study should be acknowledged. Firstly, all data was based on self-reported measures, which might introduce response bias. However, self-reported questionnaires were commonly used in large-scale epidemiological studies, and self-report of ADHD symptoms were found to be highly correlated with the results in diagnostic interviews [48]. Secondly, causal relations cannot be drawn among the psychological variables due to the cross-sectional nature of the study, which should be examined in prospective longitudinal studies in future. Thirdly, the generalizability of the findings in the current study should be taken with caution. Multi-center studies should be conducted, especially in different cultures and medical school systems.

## Conclusions

The study showed that there could be a relatively high prevalence of ADHD symptoms in Chinese medical students. Out of the two dimensions, only inattention was significantly related to life satisfaction. Resilience mediated the relationship between inattention and life satisfaction among the students. Therefore, early identification of the medical students with ADHD symptoms should be warranted. Resilience intervention programs might be undertaken to enhance life satisfaction in medical students, especially for those with inattention symptoms.

## Abbreviations

ADHD: Attention-deficit/hyperactivity disorder
ASRS: Adult ADHD Self-Report Scale
BCa 95%CI: Bias-corrected and accelerated 95% confidence interval
CD-RISC: Connor–Davidson Resilience Scale
SWLS: Satisfaction With Life Scale
WURS: Wender Utah Rating Scale
